# RADICAL: a rationally designed ion channel activated by ligand for chemogenetics

**DOI:** 10.1093/procel/pwae048

**Published:** 2024-09-03

**Authors:** Heng Zhang, Zhiwei Zheng, Xiaoying Chen, Lizhen Xu, Chen Guo, Jiawei Wang, Yihui Cui, Fan Yang

**Affiliations:** Liangzhu Laboratory, Zhejiang University Medical Center, Hangzhou 311121, China; Kidney Disease Center of the First Affiliated Hospital and Department of Biophysics, Zhejiang University School of Medicine, Hangzhou 311121, China; Department of Neurobiology, Department of Neurology of Sir Run Run Shaw Hospital, Zhejiang University School of Medicine, Hangzhou 310058, China; NHC and CAMS Key Laboratory of Medical Neurobiology, MOE Frontier Science Center for Brain Research and Brain–Machine Integration, School of Brain Science and Brain Medicine, Zhejiang University, Hangzhou 310058, China; Liangzhu Laboratory, Zhejiang University Medical Center, Hangzhou 311121, China; Kidney Disease Center of the First Affiliated Hospital and Department of Biophysics, Zhejiang University School of Medicine, Hangzhou 311121, China; Liangzhu Laboratory, Zhejiang University Medical Center, Hangzhou 311121, China; Kidney Disease Center of the First Affiliated Hospital and Department of Biophysics, Zhejiang University School of Medicine, Hangzhou 311121, China; Department of Neurobiology, Department of Neurology of Sir Run Run Shaw Hospital, Zhejiang University School of Medicine, Hangzhou 310058, China; NHC and CAMS Key Laboratory of Medical Neurobiology, MOE Frontier Science Center for Brain Research and Brain–Machine Integration, School of Brain Science and Brain Medicine, Zhejiang University, Hangzhou 310058, China; Liangzhu Laboratory, Zhejiang University Medical Center, Hangzhou 311121, China; Kidney Disease Center of the First Affiliated Hospital and Department of Biophysics, Zhejiang University School of Medicine, Hangzhou 311121, China; Department of Neurobiology, Department of Neurology of Sir Run Run Shaw Hospital, Zhejiang University School of Medicine, Hangzhou 310058, China; NHC and CAMS Key Laboratory of Medical Neurobiology, MOE Frontier Science Center for Brain Research and Brain–Machine Integration, School of Brain Science and Brain Medicine, Zhejiang University, Hangzhou 310058, China; Liangzhu Laboratory, Zhejiang University Medical Center, Hangzhou 311121, China; Kidney Disease Center of the First Affiliated Hospital and Department of Biophysics, Zhejiang University School of Medicine, Hangzhou 311121, China

## Dear Editor,

Manipulating neuronal activities by exogenous means is always much desired in the field of neuroscience and beyond. Though many optogenetics and chemogenetics tools, such as channelrhodopsins and DREADDs (Armbruster et al., 2007; Sternson and Roth, 2014), have been developed and widely used, there are several limitations in these tools. For optogenetics, the invasiveness of implanted optical fiber is unavoidable. For chemogenetics, the DREADDs are G-protein coupled receptors that rely on complex cellular signaling networks to indirectly modulate ion channels to influence neuronal activity (Armbruster et al., 2007), therefore, their pharmacokinetics are slow (Lerchner et al., 2007). Chemogenetic tools that utilize engineered chimeric ligand-gated ion channels, like the nicotinic acetylcholine receptor (nAChR), are designed to modify neuronal excitability directly (Magnus et al., 2011). However, since the nAChR is abundantly expressed in the brain, how the subunits in the engineered nAChR interact with the endogenous subunits, as well as the potential effects of these interactions, remain to be investigated. Therefore, a noninvasive chemogenetic tool that directly manipulates neuronal potential with little endogenous expression of its native form in the brain is needed.

**Figure 1.**
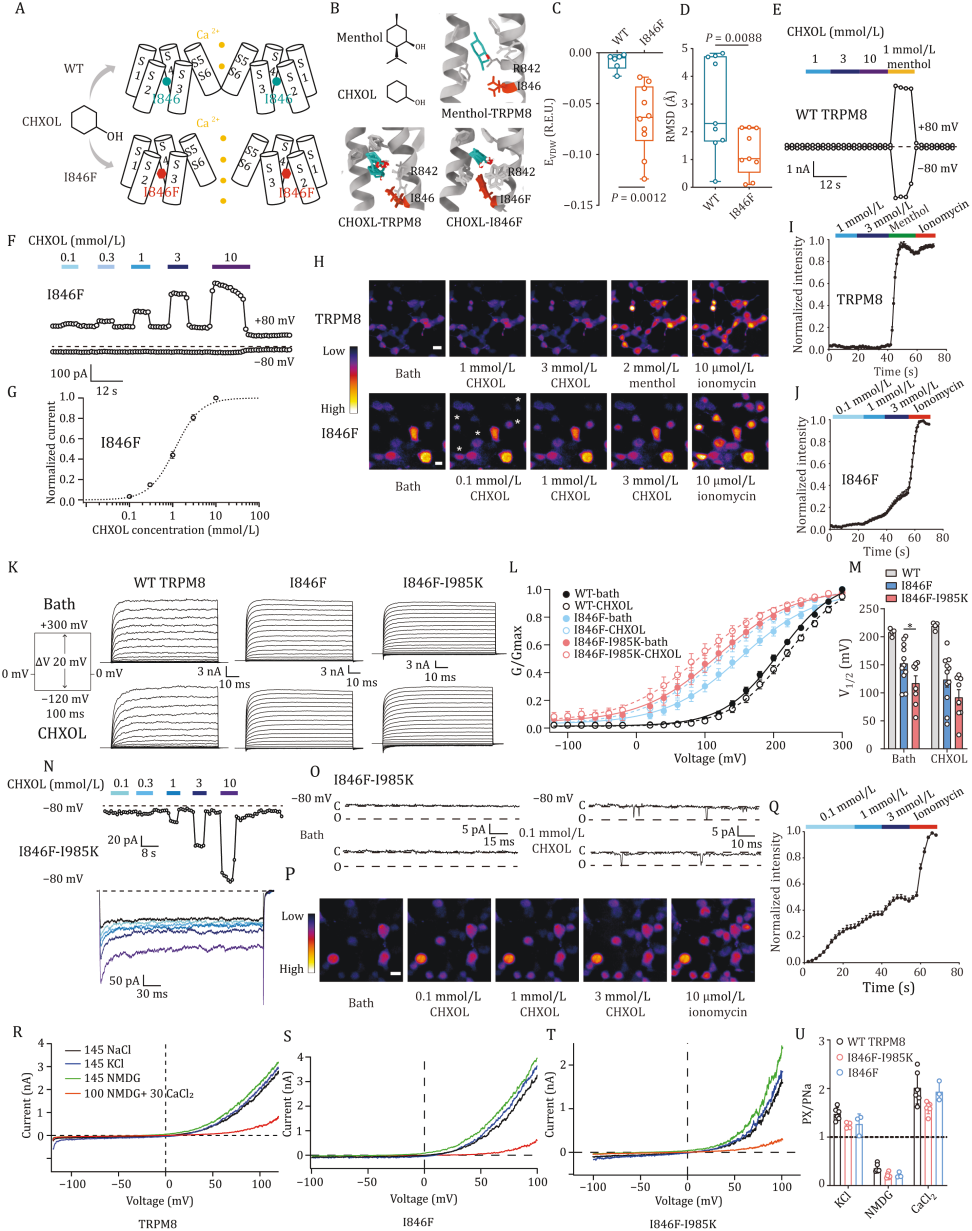
Engineering of TRPM8 channel to be activated by CHXOL and more sensitive to CHXOL. (A) The approach to engineer the TRPM8 channel to respond to CHXOL. (B) Upper left panel, the structure of menthol and CHXOL. Upper right and bottom panels, comparison of docking of menthol to wild type TRPM8, CHXOL to WT TRPM8, and CHXOL to TRPM8 I846F mutant. (C and D) Energies of VDW interactions in Rosetta energy unit (R.E.U.) and the root mean square deviation (RMSD) between CHXOL and TRPM8 or I846F mutant (*n* = 10). E, energy. Data were shown as mean ± S.E.M. Unpaired two-sided *t*-test was used to analyze statistical significance. (E) Whole-cell patch-clamp recordings of WT TRPM8 channels in response to CHOXL at the membrane potential of ±80 mV. (F) Concentration–response of CHOXL on TRPM8 I846F mutant at ±80 mV. (G) Concentration–response curve of CHOXL on TRPM8 I846F mutant (*n* = 7). (H) Top panel, calcium imaging of HEK293T cells expressing WT TRPM8 channels in response to CHXOL, menthol or 10 μmol/L ionomycin. Bottom panel, calcium imaging of HEK293T cells expressing I846F mutant in response to CHXOL or 10 μmol/L ionomycin, scale bar = 25 μm. Asterisks represent cells with a significant increase in intracellular calcium. (I) The normalized fluorescence intensity of HEK293T cells expressing WT TRPM8 channels (*n* = 14). (J) The normalized fluorescence intensity of HEK293T cells expressing I846F mutant (*n* = 8). (K) Representative currents elicited by a voltage step protocol (inset) in the presence or absence of 5 mmol/L CHXOL recorded from HEK293 cells expressing WT TRPM8 or TRPM8 mutants. (L) Normalized G-V curves for WT TRPM8 or TRPM8 mutants in the absence and presence of CHXOL (*n* = 4–11). Boltzmann equation was used to fit the G–V relationship. (M) Bar graph of *V*_1/2_ values calculated from panel (L) for WT TRPM8 or TRPM8 mutants in the absence and presence of CHXOL (*n* = 4–11; *P < 0.05 by one way ANOA). (N) Top panel, the concentration–response of CHOXL on I846F-I985K mutant at −80 mV. Bottom panel, representative current traces of I846F-I985K mutant activated by CHOXL from the top panel. (O) Representative single-channel current traces of I985K-I846F mutant in the bath and in the presence of 0.1 mmol/L CHXOL were recorded at −80 mV. O and C indicate open and closed states, respectively. (P) Calcium imaging of HEK293T cells expressing I846F-I985K mutant in response to CHXOL or ionomycin, scale bar = 25 μm. (Q) The normalized fluorescence intensity of HEK293T cells expressing I846F-I985K mutant (*n* = 19). (R–T) Representative the current of TRPM8 and I846F-I985K mutants in extracellular perfusion of 145 mmol/L NaCl, KCl, NMDG or 100 mmol/L NMDG + 30 mmol/L CaCl_2_. Voltage ramps were used to generate current–voltage curves. (U) Ion permeability ratio was calculated from reversal potentials in panel (R–T) (*n* = 3–6).

To develop such a chemogenetic tool, we targeted the transient receptor potential melastatin 8 (TRPM8), which is a calcium-permeable ion channel with limited expression regions in the brain (Jang et al., 2012; McKemy et al., 2002; Mohandass et al., 2020; Peier et al., 2002). We rationally designed a mutant TRPM8 channel to be activated by the chemical cyclohexanol (CHXOL) (Fig. 1A). CHXOL is analogous to the classic TRPM8 agonist menthol, but it lacks the isopropyl and methyl groups as compared to menthol (Fig. 1B). CHXOL (1–3 mmol/L) did not affect activities of a range of ion channels including hERG and many TRP channels (Fig. S1). Furthermore, CHXOL did not alter the resting membrane potential (RMP) or action potential in neurons and brain slices of mice (Fig. S2). As we found that the interaction between isopropyl group in menthol and residue I846 in TRPM8 is essential for binding (Fig. 1B) (Xu et al., 2020), CHXOL lacking this group did not bind well with TRPM8 as the top 10 scored CHXOL binding models with best binding energy were highly diversified in their binding configuration as shown in the molecular docking (Fig. 1B).

CHXOL cannot activate the wildtype TRPM8 even at a high concentration of 10 mmol/L in patch-clamp recordings (Fig. 1E). Therefore, we hypothesized that by increasing the sidechain bulkiness in I846, binding of CHXOL to TRPM8 mutant can be re-established to open the mutant channel. To test this hypothesis, we first docked CHXOL to the TRPM8-I846F mutant with a bulky sidechain. We observed that the top 10 scored models of CHXOL exhibited better convergence and larger binding energy (Fig. 1B–D). Indeed, in patch-clamp recordings, we found CHXOL concentration-dependently activated TRPM8-I846F with the EC_50_ of 1.13 ± 0.07 mmol/L (Fig. 1F and 1G). On the other hand, when residue I846 was mutated to residues with smaller side chains such as glycine (G, no side chain), alanine (A), and leucine (L), the mutated channels could not respond to CHXOL (Fig. S3).

However, as the current elicited by CHXOL at hyperpolarizing membrane potential of −80 mV was small (Fig. 1F), we further performed calcium imaging to test whether CHXOL can induce calcium influx. We observed that 1 mmol/L CHXOL indeed elicited calcium influx in cells expressing TRPM8-I846F (Fig. 1H and 1J), while no such increase in intracellular calcium was observed by CHXOL in cells expressing the wildtype TRPM8 (Fig. 1H and 1I). These results suggested we indeed engineered a TRPM8 mutant to respond to CHXOL as we designed.

To further improve the CHXOL sensitivity in TRPM8-I846F at the physiologically relevant hyperpolarizing membrane potential, we introduced the I985K mutation to TRPM8-I846F as this mutation enhances the voltage sensitivity of TRPM8 (Taberner et al., 2014). As expected, the G–V curve of I846F-I985K mutant showed a dramatic left shift with half-maximal activation voltage (*V*_1/2_) of 117.2 ± 13.2 mV as compared with WT (208.2 ± 3.4 mV) or I846F mutant (152.4 ± 10.8 mV). And the addition of CHXOL decreased the *V*_1/2_ to 123.6 ± 13.5 mV for I846F mutant and 91.9 ± 13.7 mV for I985K-I846F (Fig. 1K–M). As a result, the TRPM8-I846F-I985K double mutant exhibited a much larger current activated by CHXOL at −80 mV (Fig. 1N). Though the EC_50_ to CHXOL on TRPM8-I846F-I985K double mutant remains unchanged at +80 mV (1.17 ± 0.11 mmol/L) as compared to TRPM8-I846F mutant, these results suggested that the introduction of I985K indeed enhanced the voltage sensitivity in this channel. In single-channel recordings, CHXOL also elicited opening events in the double mutant at −80 mV (Fig. 1O). In calcium imaging, 100 µmol/L CHXOL, which barely induced calcium influx with TRPM8-I846F, elicited robust calcium signal in cells transfected with TRPM8-I846F-I985K mutant (Fig. 1P and 1Q). Moreover, prolonged exposure of CHXOL over 50 s almost decreased the intracellular calcium fluorescence to baseline (Fig. S4A and S4B). Such a decrease in calcium fluorescence was not due to photobleaching of the loaded Fluo-4 AM dyes because we observed a higher fluorescence intensity upon the application of ionomycin. In addition, the presence of 3 mmol/L CHXOL did not increase the cell death ratio in HEK293T cells expressing WT TRPM8 channels or I846F-I985K mutants (Fig. S4C and S4D), indicating the extent of CHXOL-induced activation of the rationally designed ion channel activated by ligand (RADICAL) was insufficient to induce cell death. These results suggested the influx of calcium was controllable and the amount of Ca^2+^ influx was limited. Moreover, the calcium selectivity of the TRPM8-I846F-I985K mutant was maintained as the wildtype TRPM8 channel (Fig. 1R–U).

We further test the ligand activation and temperature activation on the TRPM8-I846F-I985K mutant. I846F-I985K mutant and WT TRPM8 showed similar response to icilin with EC50 of 53.8 ± 15.3 nmol/L and 51.4 ± 12.9 nmol/L, respectively (Fig. S5A and S5B). In addition, we also calculated the change of enthalpic (Δ*H*) and entropic (Δ*S*) due to the temperature-driven transition of the TRPM8 channel. Δ*H* and Δ*S* exhibited similar behavior among WT TRPM8, I846F and I846F-I985K mutants, suggesting the temperature-related property of RADICAL remains unchanged (Fig. S5D–G).

**Figure 2.**
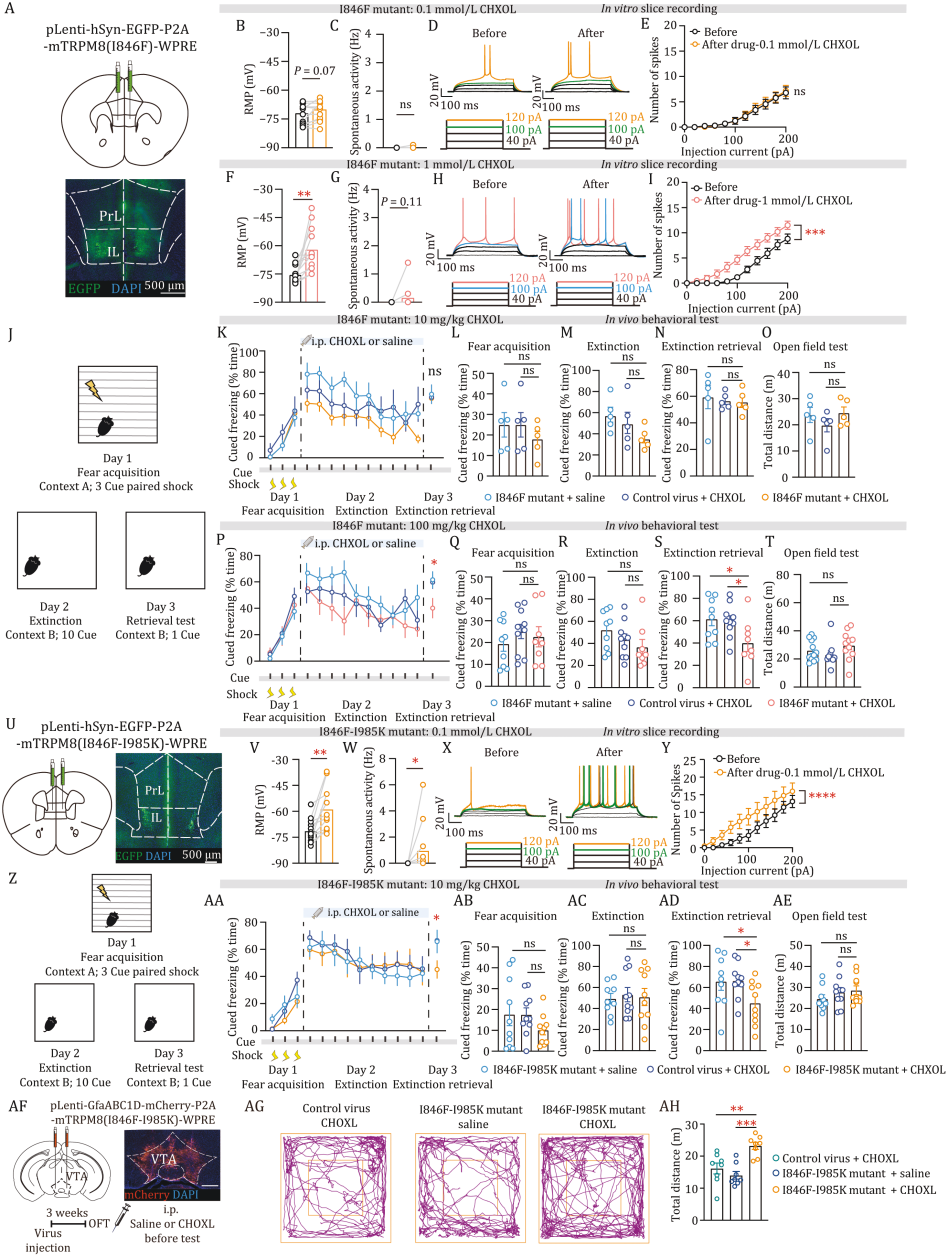
Activation of the CHXOL-TRPM8 system in infralimbic cortex neurons and ventral tegmental area (VTA) astrocytes by viral injection enhanced retrieval of fear extinction memory and locomotor behavior in mice, respectively. (A) Illustration (top) and a representative image (bottom) of viral delivery in IL. (B–E) Electrophysiological results of 0.1 mmol/L CHXOL on I846F-mutant expressing IL neurons. (B) RMP, *n* = 13, mice = 7, Paired *t*-test, *P* = 0.0728; (C) spontaneous activity, *n* = 13, mice = 7, Paired *t*-test, *P* = 0.3370; (D) Raw traces showed voltage responses to a series of 500 ms current pulses from 0 to 200 pA with 20-pA steps; (E) number of induced APs with 20-pA current steps, *n* = 13, mice = 7, Paired *t*-test, *P* = 0.0977. (F–G) Statistics show significant depolarization effects of 1 mmol/L CHXOL on resting membrane potential (RMP) (F, *n* = 12, mice = 7, Paired *t*-test, *P* = 0.0055, *t* = 3.447, df = 11) but not on the spontaneous activity (G, *n* = 12, mice = 7, Paired *t*-test, *P* = 0.1820, *t* = 1.425, df = 11) in I846F-mutant expressing mice. (H–I), Current–voltage relationship before and during 1 mmol/L CHXOL perfusion. Raw traces of panel H showed voltage responses to a series of 500 ms current pulses from 0 to 200 pA with 20-pA steps. The number of induced APs with 20-pA current steps was significantly increased after CHXOL perfusion (I, *n* = 11, mice = 7, paired *t*-test, *P* = 0.0001, *t* = 5.964, df = 10). (J) Experimental design. Mice fear conditioned to three tone-shock pairings (0.6 mA/2-s shock) on day 1; extinction on day 2; CHXOL or saline administration 30 min before extinction test on day 2; retrieval test on day 3. (K–O) 10 mg/kg CHXOL showed no effect on extinction retrieval (*n* = 8–10; Fear acquisition test, One-way ANOVA, *P* = 0.5795, *P*_(I846F-(I985K)-mutant + saline vs I846F-(I985K)-mutant+CHXOL)_ = 0.3729, *P*_(control virus + CHXOL vs I846F-(I985K)-mutant + CHXOL)_ = 0.3729; extinction test, one-way ANOVA, *P* = 0.1563, *F*(2, 12) = 2.176, *P*_(I846F-(I985K)-mutant + saline vs I846F-(I985K)-mutant+CHXOL)_ = 0.0623, *P*_(control virus + CHXOL vs I846F-(I985K)-mutant + CHXOL)_ = 0.2062; extinction retrieval test, one-way ANOVA, *P* = 0.8901, *F*(2, 12) = 0.1176, *P*_(I846F-(I985K)-mutant + saline vs I846F-(I985K)-mutant+CHXOL)_ = 0.6494, *P*_(control virus + CHXOL vs I846F-(I985K)-mutant + CHXOL)_ = 0.9084; open field test, one-way ANOVA, *P* = 0.4121, *F*(2, 12) = 0.9552, *P*_(I846F-(I985K)-mutant + saline vs I846F-(I985K)-mutant + CHXOL)_ = 0.8347, *P*_(control virus + CHXOL vs I846F-(I985K)-mutant + CHXOL)_ = 0.2216). (P–T) 100 mg/kg CHXOL activation of I846F-mutant-expressing IL during extinction training did not affect the expression of freezing but facilitated extinction retrieval on the following day without altering locomotion in an open-field test (*n* = 8–10; fear acquisition test, one-way ANOVA, *P* = 0.5555, *F*(2, 24) = 0.6025, *P*_(I846F-mutant + saline vs I846F-mutant + CHXOL)_ = 0.5612, *P*_(control virus + CHXOL vs I846F-mutant + CHXOL)_ = 0.6507; extinction test, one-way ANOVA, *P* = 0.2204, *F*(2, 24) = 1.612, *P*_(I846F-mutant + saline vs I846F-mutant + CHXOL)_ = 0.0871, *P*_(control virus + CHXOL vs I846F-mutant + CHXOL)_ = 0.4323; extinction retrieval test, one-way ANOVA, *P* = 0.0427, *F*(2, 24) = 3.605, *P*_(I846F-mutant + saline vs I846F-mutant + CHXOL)_ = 0.0230, *P*_(control virus + CHXOL vs I846F-mutant + CHXOL)_ = 0.0319; open field test, one-way ANOVA, *P* = 0.2385, *F*(2, 30) = 1.504, *P*_(I846F-mutant+saline vs I846F-mutant+CHXOL)_ = 0.3378, *P*_(control virus + CHXOL vs I846F-mutant + CHXOL)_ = 0.0940). (U) Illustration (top) and a representative image (bottom) of viral delivery in IL. (V and W) Statistics show significant depolarization effects of CHXOL (0.1 mmol/L) on RMP (V, *n* = 12, mice = 3, Paired *t*-test, *P* = 0.0022, *t* = 3.963, df = 11) and the spontaneous activity (W, *n* = 12, mice = 3, Wilcoxon test, *P* = 0.0312, *W* = 21) in I846F-I985K-mutant expressing mice. (X and Y) Current–voltage relationship before and during 0.1 mmol/L CHXOL perfusion. Raw traces showed voltage responses to a series of 500 ms current pulses from 0 to 200 pA with 20-pA steps. Green and yellow traces indicate APs induced by 100 pA and 120 pA injected current. The number of induced APs with 20-pA current steps was significantly increased after CHXOL perfusion (Y, *n* = 9, mice = 3, Paired *t*-test, *P* < 0.0001, *t* = 8.099, df = 10). (Z-AE) Experimental design. Mice fear conditioned to three tone-shock pairings (0.6 mA/2-s shock) on day 1; extinction on day 2; 10 mg/kg CHXOL or saline administration 30 min before extinction test on day 2; retrieval test on day 3. (Z, mice_I846F-(I985K)-mutant + saline_ = 10, mice_control virus + CHXOL_ = 10, mice_I846F-(I985K)-mutant + CHXOL_ = 10; AA–AC, mice_I846F-(I985K)-mutant + saline_ = 9, mice_control virus + CHXOL_ = 10, mice_I846F-(I985K)-mutant + CHXOL_ = 10). AD–AE, CHXOL activation of I846F-I985K-mutant-expressing IL during extinction training did not affect the expression of freezing but facilitated extinction retrieval at the following day without altering locomotion in the open-field test (fear acquisition test, one-way ANOVA, *P* = 0.2994, *F*(2, 27) = 1.261, *P*_(I846F-(I985K)-mutant + saline vs I846F-(I985K)-mutant + CHXOL)_ = 0.1788, *P*_(control virus + CHXOL vs I846F-(I985K)-mutant + CHXOL)_ = 0.1817; extinction test, one-way ANOVA, *P* = 0.9230, *F*(2, 26) = 0.08042, *P*_(I846F-(I985K)-mutant + saline vs I846F-(I985K)-mutant + CHXOL)_ = 0.8710, *P*_(control virus + CHXOL vs I846F-(I985K)-mutant + CHXOL)_ = 0.8123; extinction retrieval test, one-way ANOVA, *P* = 0.0514, *F*(2, 26) = 3.335, *P*_(I846F-(I985K)-mutant + saline vs I846F-(I985K)-mutant + CHXOL)_ = 0.0403, *P*_(control virus + CHXOL vs I846F-(I985K)-mutant + CHXOL)_ = 0.0304; open field test, one-way ANOVA, *P* = 0.3780, *F*(2, 26) = 1.010, *P*_(I846F-(I985K)-mutant + saline vs I846F-(I985K)-mutant + CHXOL)_ = 0.1864, *P*_(control virus + CHXOL vs I846F-(I985K)-mutant + CHXOL)_ = 0.7729). (AF) Schematic of the experimental design for testing locomotion in mice that express mTRPM8 (I846F-I985K) in VTA astrocytes, scale bar = 500 µm. (AG) Representative examples of locomotion in the open-field test (OFT). (AH) Statistics of total distance traveled in OFT 30 min after i.p. CHXOL or saline injection in mice which were injected with pLenti-mTRPM8 (I846F-I985K) or its control virus in VTA (OFT, *n* = 8 mice for each group, one-way ANOVA, *P* = 0.0003, *F* = 12.42, *P*_(I846F-(I985K)-mutant + saline vs I846F-(I985K)-mutant + CHXOL)_ = 0.0003, *P*_(control virus + CHXOL vs. I846F-(I985K)-mutant + CHXOL)_ = 0.004).

We next employed virus-based delivery techniques to assess the *in vivo* effects of activating the CHXOL-TRPM8 mutant system in mice. We used lentivirus, which allows packaging of plasmid with 4.5–6 bp in length, together with the human synapsin promoter to deliver eGFP tagged mutant TRPM8 channel specifically in neurons (both pLenti-hSyn-TRPM8 (I846F) and pLenti-hSyn-TRPM8 (I846F-I985K)) (Fig. S6). We chose to express the virus in the infralimbic cortex (IL) (Fig. S7), which is necessary for the retrieval of fear memory extinction in rodents (Duvarci and Pare, 2014; Orsini and Maren, 2012). Twenty-one days after bilateral injection into the IL, pLenti-mediated viral transfection led to the expression of mTRPM8 (I846F) in neurons throughout the IL (Fig. 2A). To confirm the efficacy of the CHXOL-mTRPM8 I846F mutant system, we performed whole-cell patch clamp recordings from virally transfected IL neurons (Fig. 2B–I). We found that while 0.1 mmol/L CHXOL did not affect any physiological characteristics of the viral transfected neurons (Fig. 2B–E), 1 mmol/L CHXOL depolarized RMP, increased the intrinsic excitability on pLenti-hSyn-TRPM8 (I846F) transfected neurons (Fig. 2F–I).

To assess if *in vivo* activation of the CHXOL-TRPM8 (I846F) system affects IL-related behavioral consequences, we injected CHXOL (i.p. 10 mg/kg and 100 mg/kg) or the vehicle control in mice expressing pLenti-hSyn-TRPM8 (I846F). As expected, in the fear conditioning test (Fig. 2J–T), activation of IL TRPM8 (I846F) transfected neurons by 100 mg/kg but not 10 mg/kg CHXOL enhanced the retrieval of fear extinction memory without affecting locomotion levels compared to the virus and vehicle controls (Fig. 2J–T).

As the TRPM8-I846F-I985K double mutant exhibits a higher sensitivity to CHXOL (Fig. 1N–Q), we further tested the *in vivo* efficacy of pLenti-hSyn-TRPM8 (I846F-I985K) system (Fig. 2U). We found that a much lower concentration of CHXOL (0.1 mmol/L) significantly depolarized RMP, increased the intrinsic excitability on pLenti-hSyn-TRPM8 (I846F-I985K) transfected neurons (Fig. 2V–Y). In the fear conditioning test (Fig. 2Z), a lower concentration of 10 mg/kg CHXOL significantly enhanced the retrieval of extinction memory of TRPM8 (I846F-I985K) mice compared to the virus and vehicle controls (Fig. 2AA–AE).

Furthermore, we also tested the *in vivo* efficacy of RADICAL in glial cells in the ventral tegmental area (VTA). The astrocytes in VTA played a central role in the control of locomotor activity (Requie et al., 2022). We used lentivirus, together with the astrocyte-specific GfaABC1D promoter to deliver mCherry tagged mutant TRPM8 channel specifically in VTA astrocytes. After bilateral VTA injection of pLenti-GfaABC1D-mCherry-P2A-mTRPM8 (I846F-I985K)-WPRE virus, locomotion of mice was tested (Fig. 2AF). As we expected, 10 mg/kg CHXOL induced locomotor hyperactivity in TRPM8 (I846F-I985K) mice as compared to control mice (Fig. 2AG–AH).

Therefore, we have developed a rationally designed ion channel activated by ligand (RADICAL) for chemogenetics manipulation of animal behaviors. As RADICAL is based on calcium permeable TRPM8-I846F-I985K mutant, neuronal processes where elevation of intracellular calcium level is involved, such as learning and memory, can be tuned by this tool. TRPM8 channel is barely expressed in the brain (Jang et al., 2012; McKemy et al., 2002; Peier et al., 2002), so virus injection and expression of our RADICAL tool in the brain are expected to less perturb the subunit assembly and physiological function of endogenous ion channels.

Though our RADICAL is currently hosted on a lentiviral vector, such a system is compatible with the Cre recombinase (Nie et al., 2018; Sumegi et al., 2012). It is feasible to specifically express RADICAL in different cell types of Cre mice. We acknowledge that due to the size of RADICAL being too large, it cannot be packaged into an adeno-associated virus (AAV) vector so the application of the current version of RADICAL may be partially limited. Therefore, to broaden the usage of RADICAL we are dedicated to engineer an improved version of this chemogenetics tool in future, which can be packaged into an AAV vector by decreasing the size of TRPM8 without changing its channel activity.

## Supplementary data

Supplementary data is available at https://doi.org/10.1093/procel/pwae048.

pwae048_suppl_Supplementary_Materials

## Footnotes

We are grateful to Ms Yiqin Liu of Zhejiang University and our lab members for their assistance and discussion.

H.Z., X.C., L.X., and J.W. conducted the *in vitro* experiments including patch-clamp recordings, calcium imaging and molecular docking; Z.W. and C.G conducted animal behavior experiments and patch-clamp recordings on brain slices. F.Y. and Y.C. conceived and supervised the project and prepared the manuscript; H.Z., Z.W., Y.C. and F.Y. participated in data analysis and manuscript writing.

The authors declare no conflicts of interest.

This work was supported by funding from the National Natural Science Foundation of China (32122040 and 31971040 to F.Y.; 32071017 and 31922031 to Y.C.); Zhejiang Provincial Natural Science Foundation of China (LR20C050002 to F.Y.); China Postdoctoral Program for Innovative Talents (BX20230323 to H.Z.); China Postdoctoral Science Foundation (2024M752858 to H.Z.); The Fundamental Research Funds for the Central Universities (226-2022-00227 to F.Y.; 226-2022-00149 to Y.C.); Shenzhen-Hong Kong Institute of Brain Science-Shenzhen Fundamental Research Institutions (NYKFKT2019001 to Y.C.); The Fundamental Research Funds for the Central Universities (226-2022-00227 to F.Y.).

All animal studies and experimental procedures were approved by the Animal Care and Use Committee of the animal facility at Zhejiang University. Informed consents were obtained from all the authors. The authors declare their agreement to publish.

All data needed to evaluate the conclusions in the paper are presented in the paper. Additional data is available from authors upon request.
