# Supplementary material for: RADICAL: a rationally designed ion channel activated by ligand for chemogenetics
Source: Protein Cell. 2024 Sep 3;16(2):136–42. doi: 10.1093/procel/pwae048 (PMC11786721; doi:10.1093/procel/pwae048)
Supplement: pwae048_suppl_Supplementary_Materials [file pwae048_suppl_supplementary_materials.pdf]

Supplemental materials for

RADICAL: a rationally designed ion channel activated by ligand for chemogenetics

Heng Zhang [1,2,5], Zhiwei Zheng [3,4,5], Xiaoying Chen [1,2], Lizhen Xu [1,2], Chen Guo [3,4], Jiawei Wang [1,2], Yihui Cui [3,4]*, Fan Yang [1,2]*

[1] Liangzhu Laboratory, Zhejiang University Medical Center, Hangzhou, Zhejiang 311121, China.

[2] Kidney Disease Center of the First Affiliated Hospital and Department of Biophysics, Zhejiang University School of Medicine, Hangzhou, Zhejiang Province, China

[3] Department of Neurobiology, Department of Neurology of Sir Run Run Shaw Hospital, Zhejiang University School of Medicine, 310058, Hangzhou, China

[4] NHC and CAMS Key Laboratory of Medical Neurobiology, MOE Frontier Science Center for Brain Research and Brain-Machine Integration, School of Brain Science and Brain Medicine, Zhejiang University, 310058 Hangzhou, China

[5] These authors contributed equally to this work

*Correspondence should be addressed to:

Fan Yang (fanyanga@zju.edu.cn), Yihui Cui (yihuicui@zju.edu.cn)

Materials and methods

Chemicals

Cyclohexanol (CHOXL) were purchased from Sigma Aldrich (MCE) and Sangon Biotech. Menthol was purchased from TCI Shanghai, Capsaicin was purchased from MCE, GSK1016790A was purchased from Topscience, AITC, 2-APB were purchased from Sigma-Aldrich, and A-967079 was purchased from Tocris. All compounds except additional statement were dissolved in DMSO.

Animals

Male adult (7-15 weeks of age) C57BL/6J mice (SLAC) were used for this project. Mice were housed 4-5 per cage under a 12-h light-dark cycle (light on from 7 a.m. to 7 p.m.) with ad libitum food and water. All animal studies and experimental procedures were approved by the Animal Care and Use Committee of the animal facility at Zhejiang University.

Cell culture

HEK293T cells are cultured using DMEM medium contained 10% FBS in a cell incubator with 5% CO_2 at 37 °C. Plasmids of murine TRPV1, TRPV2, TRPV3, TRPV4, TRPM8 and human TRPA1, Kv4.2, Nav1.7 and hERG channels are labeled with YFP or GFP as indicators for subsequent electrophysiological recordings. cDNA constructs of ion channels or TRPM8 mutants were transiently transfected with Lipofectamine 2000 (Invitrogen, Carlsbad, CA). 18-24 h after transient transfection, electrophysiological recordings were performed.

Site mutations of TRPM8 channel were generated by Fast Mutagenesis Kit (Takara Bio). All mutations were confirmed by sequencing.

Molecular docking of CHOXL to TRPM8 channel and generation of I846F mutant model

The molecular docking was performed as previously described(Yang et al., 2015). In brief, RosettaLigand procedure of Rosetta program suite version 2019 was used to perform molecular docking of CHOXL to TRPM8 and generate TRPM8 mutant model (Meiler and Baker, 2006) , and Monte Carlo algorithm was employed for sampling. The three-dimensional 3D conformation of CHOXL was generated by Frog2 server (Miteva et al., 2010). The structure of TRPM8 (PDB ID: 6NR3) was relaxed by Relax application in Rosetta program suite.

The relaxed structure of wildtype TRPM8 was used as input file to generate model of TRPM8 I846F mutant in Rosetta Backrub application (Smith and Kortemme, 2008). 100 models were generated and the model with lowest energy was selected for further relax. The best structure model with lowest energy of I846F mutants was relaxed as described above. 100 relaxed models were generated and models with the lowest energy were chosen as candidates for the docking step.

CHOXL was put in the menthol-binding pocket of WT TRPM8 and I846F mutant, the top 10 models with lowest binding energy from 30000 generated models were chosen for further analysis. Rosetta's Residue_energy_breakdown application was used to extract binding energies between CHXOL and TRPM8.

Electrophysiology

Patch-clamp recordings were carried out with a HEKA EPC10 amplifier controlled by PatchMaster software (HEKA). Patch pipettes were prepared from borosilicate glass and fire-polished to resistance of 3~8 MΩ by P-97 puller. For whole-cell recordings of TRPM8 channel and AISC channels, bath solution contained 145 mM NaCl, 2 mM KCl, 2 mM $MgCl_2$, 1.5 mM $CaCl_2$, 10 mM HEPES, 10 mM glucose were adjusted to pH 7.2 ~ 7.4 with NaOH; pipette solution contained 135 mM CsCl, 1 mM $MgCl_2$, 2 mM $CaCl_2$, 10 mM HEPES, 5 mM EGTA were adjusted to pH 7.2 ~ 7.4 with CsOH. For solution with pH 5.0, HEPES was replaced by MES. To obtain icilin-induced TRPM8 current, a solution containing 135 mM NaCl, 0.5 mM $CaCl_2$, 10 mM glucose and 3 mM HEPES was used as pipette and bath solution. Whole-cell recordings were performed at ±80 mV. Current was sampled at 10 kHz and filtered at 2.9 kHz.

For cation permeability experiment, the bath solution and pipette solution contained 145 mM NaCl, 10 mM HEPES and 10 mM glucose (pH 7.4 with NaOH). After the whole-cell configuration was obtained in bath solution, the external Na^+ was changed to 145 mM K^+ and 145 mM $NMDG^+$ (pH 7.4). For Ca^{2+} permeability measurement, external Na+ was replaced with 100 mM NMDG and 30 mM $CaCl_2$. Cation permeability was estimated by the shift of reversal potential relative to Na^+, which can be obtained from voltage ramp recordings (voltage ramp from -120 mV to 120 mV in 360 ms). Monovalent cation permeability and Ca^{2+} permeability was calculated from the equation (1) and (2), respectively (Valera et al., 1994),(Xu et al., 2002).

$$P_X/P_{Na} = \{[Na^+]_o/[X]_o\}\{\exp((F/RT)(V_X - V_{Na}))\} \quad (1)$$

$$P_{Ca}/P_{Na} = \{[Na^+]_o/4[Ca^{2+}]_o\}\{\exp((F/RT)(V_{Ca} - V_{Na}))\}\{1+ \exp(FV_{Ca}/RT)\} \quad (2)$$

where [X]o is defined as the extracellular concentration of the monovalent cations, *P* is defined as the permeability of the ion. *F* is Faraday's constant, *R* is the gas constant, *T* is temperature in Kelvins and *V* is the reversal potential for the cation ions.

For whole-cell recordings of TRPV1, TRPV2, TRPV3, TRPV4, and TRPA1 channels, both bath and pipette solutions contained (in mM): 130 NaCl, 10 glucose, 0.2 EDTA, and 3 HEPES (pH 7.2 ~ 7.4, adjusted with NaOH). Whole-cell recordings were performed at ±80 mV. Current was sampled at 10 kHz and filtered at 2.9 kHz. For whole-cell recordings of Nav1.7 channels, bath solution contained (in mM): 145 NaCl, 2 KCl, 2 $MgCl_2$, 1.5 $CaCl_2$, 10 HEPES, 10 glucose (pH 7.2 ~ 7.4, adjusted with NaOH); pipette solution contained (in mM): 140 CsF, 10 NaCl, 1 EGTA and 10 HEPES (pH 7.3, adjusted with CsOH). Current traces were evoked by a 200 ms step depolarization to −10 mV or 0 mV from a holding potential of −80 mV every 2 s to avoid the fast desensitization of Nav channels. For whole-cell recordings of Kv4.2 channels, bath solution contained (in mM): 145 NaCl, 2 KCl, 2 $MgCl_2$, 1.5 $CaCl_2$, 10 HEPES, 10 glucose (pH 7.2 ~ 7.4, adjusted with NaOH); pipette solution contained (in mM): 135 KCl, 2 $MgCl_2$, 1 EGTA, 10 HEPES and 2 Na_2ATP (pH 7.3, adjusted with KOH). To obtain the current of Kv4.2 channel, the membrane potential was clamped at -60 mV for 10 ms and switched to voltage stepping from -60 mV to +60 mV with a 20-mV interval for 300 ms, and then switched to -60 mV for 10 ms.

Currents were sampled at 10 kHz and filtered at 2.9 kHz.

All recordings were performed at room temperature (25 °C). A gravity-driven system (RSC-200, Bio-Logic) with freely rotated perfusion tubes was used for the perfusion of peptide or ligands. Bath and ligand-contained solutions were delivered through separate tubes to minimize the mixing of solutions. Patch pipette holding cells was placed in front of the perfusion tube outlet for perfusion.

Calcium imaging

Calcium imaging was conducted as previously described (Zhang et al., 2023). In brief, HEK293T cells expressing TRPM8 or TRPM8 mutants were incubated with 2 μM Fluo-4 AM (Thermo Fisher) in 2 mM calcium Ringer's solution (140 mM NaCl, 5 mM KCl, 2 mM $MgCl_2$, 2 mM $CaCl_2$, 10 mM glucose, and 10 mM HEPES, pH 7.4). Fluorescence images of HEK293T cells were acquired with Nikon Eclipse Ti2 microscope with optiMOS charge-coupled device camera controlled by the Ocular Software (Molecular Devices). Fluo-4 AM was excited at 500/20-nm excitation, and fluorescence emission was detected at 535/30-nm. Fluorescence images were analyzed with Fiji software and Office Excel.

Cell death assay

Cell death was Cell death assay was performed as previous described (Zhang et al., 2019; Zhang et al., 2023). In brief, HEK293T cells were seeded in six-well plates and randomly divided into different groups after transfection. Then CHXOL was incubated for 18 h before the determination of cell death. Hoechst 33258 (Sangon

Biotech) was used to labeled all cell nuclei and propidium iodide (PI) (Sangon Biotech) was used to labeled nuclei of dead cells. In each group, five images were randomly taken under a microscope (STELLARIS8, Leica, German). Cell death ratio was represented by the average of the percentage of dead cells (PI-labeled cells over Hoechst-labeled cells) from five images. Fiji software was used to count cells.

Calculation of ΔH and ΔS

Calculation of ΔH and ΔS was performed as previously described(Yang et al., 2020; Xu et al., 2021). To calculate the change of enthalpic (ΔH) and entropic (ΔS) due to the temperature-driven transition of TRPM8, we constructed Van't Hoff plots and fitted them to the equation $\ln Keq = -\Delta H/RT + \Delta S/R$, whereby R represents the gas constant, T represents the temperature in Kelvin, Keq represents the equilibrium constant measured from the cold-driven TRPM8 open probability, $Keq= Po/(1-Po)$. The TRPM8 open probability induced by cold was calculated by noise analysis.

Stereotaxic Surgery and Virus Injection

For Stereotaxic surgery, mice (7-8 weeks) were anaesthetized with Pentobarbital NEMBUTAL (100 mg/kg, Sigma-Aldrich). Viruses were bilaterally injected by a glass pipette mounted on a stereotactic frame into IL (±0.30 mm ML, +1.95 mm AP, -2.30 mm DV) and VTA (±0.5 mm ML, -3.04 mm AP, -4.30 mm DV). Volumes of virus were 1 uL per side, infused at a rate of 100 nL/min. The injection pipette was withdrawn from the brain 10 min after the infusion. After surgery, mice were recovered from anesthesia on a heat pad. The viral injection sites were verified by

post immunohistochemistry experiment as we demonstrated with schematics in Fig. S4.

Fear conditioning test

3 weeks after surgery, all mice were handled for three days before behavioral assays for 5 min per day. For cued fear conditioning experiments, mice were placed in a chamber with a grid floor connected to a shock generator (Coulbourn instruments). Three minutes after being placed in the chamber, mice were exposed to 30-s tones (3 kHz, 80 dB) co-terminating with a 2-s foot shock (0.6 mA) for three times at pseudo-random inter-trial intervals (~2 min average). The chamber was cleaned with 75% ethanol at the end of each trial. The next day for fear extinction, mice were placed in a different context (floor and walls were changed and white vinegar was sprayed in the chamber). First, mice were injected (i.p.) with CHXOL (100 mg/kg or 10 mg/kg) or saline before extinction test. After 30 min, mice were exposed to 10 tones to undergo fear extinction. On day 3, mice were placed in the same environment as in day 2 and were exposed to one presentation of the tone. Behavior was recorded using digital video cameras. Freezing was scored by the fear conditioning software (FreezeFrame).

Open field test

Mice were placed in the center of an area (45 cm × 45 cm × 45 cm) in a room with dim light for 10 min. A video camera positioned directly above the arena was used to track the movement of each animal (Any-maze, Stoelting). For mice with injection of pLenti-GfaABC1D-mCherry-P2A-mTRPM8(I846F-I985K)-WPRE virus in VTA

astrocytes, mice received an injection of CHXOL (100 mg/kg or 10 mg/kg) 30 minutes before the beginning of the test.

Slice preparation

Mice were anaesthetized with 1% pentobarbital NEMBUTAL (100 mg/kg), perfused with 200 ml ice-cold artificial cerebrospinal fluid (ACSF, oxygenated with 95% O_2, 5% CO_2) containing (in mM): 210 sucrose, 125 NaCl, 2.5 KCl, 25 $NaHCO_3$, 1.25 NaH_2PO_4, 1 $MgCl_2$, 1 $CaCl_2$, 25 glucose and 1 sodium pyruvate. After decapitation, the brains were transferred rapidly into ice-cold oxygenated ACSF. 300 mm thickness coronal slices, or sagittal slices if specified, containing the IL were sectioned in cold ACSF with a Leica VT1200S vibratome, and incubated in a recovery chamber containing ASCF (in mM): 125 NaCl, 2.5 KCl, 25 $NaHCO_3$, 1.25 NaH_2PO_4, 1 $MgCl_2$, 1 $CaCl_2$, 25 glucoses at 32 C. ACSF was continuously gassed with 95% O_2, 5% CO_2. Slices were allowed to recover for at least 1 h before recordings and then transferred to room temperature.

***In vitro* electrophysiology**

Whole cell patch clamp recordings were made from neurons in the IL under an upright microscope (BX51WI, Olympus). All recordings were performed at 34 °C using a temperature control system and slices were continuously superfused at 2-3 mL/min with the extracellular solution. The patch pipettes were pulled with a pipette puller (PC-100, Narishige) from borosilicate glass (Sutter Instrument) and had a resistance of 5-6 MΩ. Electrophysiological recordings were made using a MultiClamp

700B amplifier and pCLAMP 10.6 software (Axon Instruments). Signals were amplified, filtered at 2 kHz and sampled at 10 kHz using Digidata 1550B. The series resistance and capacitance were compensated automatically after a stable Gigaseal was formed. Experiments were discarded if a change in series resistance was above 20%.

Patch pipettes were filled with internal solution containing (in mM) 127 K-gluconate, 13 KCl, 4 Mg_3-ATP, 0.3 Na_3-GTP, 0.3 EGTA, 10 HEPES and 10 Na-phosphocreatine (pH = 7.25). The external ACSF solution contained (in mM) 125 NaCl, 2.5 KCl, 25 $NaHCO_3$, 1.25 NaH_2PO_4, 1 $MgCl_2$, 1 $CaCl_2$ and 25 glucoses. The resting membrane potential (RMP) of a neuron was obtained under current clamp (I = 0 pA), and the action potential (AP) was measured by injecting a series of current pulses (500 ms duration, -100 to 200 pA intensity with an increment of 20 pA). CHXOL (0.1 mM or 1 mM) was dissolved in DMSO and added in ACSF with a final concentration of 0.02%. The effect of CHXOL was recorded after 4 min of drug perfusion.

Statistical analysis

All statistical data are shown as mean ± S.E.M. Paired t-test was used to analyze the statistical significance between two groups in GraphPad Prism 7.0 software (GraphPad Inc., La Jolla, CA). For more than two groups, one-way ANOVA was used. n.s. indicates no significance. *, **, and *** indicate $p < 0.05$, $p < 0.01$, and $p < 0.001$, respectively. EC50 was obtained from the fitting curves to the concentration-response relationship using Hill Equation in Igor Pro 5.0 software.

Supplementary Figure Legends

Fig. S1 Selectivity of CHXOL on ion channels

A-K, The effect of 1 mM or 3 mM CHXOL on TRPV1, TRPV2, TRPV3, TRPV4, TRPA1, Kv4.2, ASIC1a, ASIC2a, ASIC3, Nav1.7 and hERG channels. l, bar graph of activation of CHXOL on these channels. (n = 3-8).

Fig. S2 CHOXL did not affect RMPs and intrinsic excitability of IL neurons

A-C, Resting membrane potential (RMP) of IL neuron after perfusion with bath or 10 mM CHXOL. (**A**, n = 6, mice = 2, paired t-test, p > 0.999; **B-C**, n = 5, mice = 2, Wilcoxon test, P = 0.0635).

Fig. S3 The effect of CHOXL on TRPM8 I846 mutants with different sizes of side chain at the membrane potential of ±80 mV.

Fig. S4 CHXOL induced limited calcium influx by activating I846F-I985K mutant. **A**, Fluorescence images of HEK293T cells expressing I846F-I985K mutant in response to CHXOL or ionomycin, scale bar = 25 μm. **B**, The normalized fluorescence intensity of HEK293T cells expressing I846F-I985K mutant (n = 4)**. C,** Hoechst and PI staining of HEK293T cells expressing WT TRPM8 channel or I846F-I985K mutant in the presence or absence of CHXOL. Hoechst labelled nuclei of all cells and PI labelled nuclei of dead cells, scale bar=100 μm. **D**, Cell death ratio calculated from panel **c**.

Fig. S5 Concentration-response of icilin on WT TRPM8 and I846F-I985K mutant. **A** and **B**, Concentration-response of CHOXL on WT TRPM8 channel and

TRPM8 I846F-I985K mutant at ±80 mV, respectively. **C**, Concentration-response curves of CHOXL on WT TRPM8 channel and TRPM8 I846F-I985K mutant (n = 6). **D**, Representative cold activation of TRPM8 and TRPM8 mutants. **E**, Van't Hoff plots for the cold-activated TRPM8 currents shown in **D**. Dotted lines represent fits of the Van't Hoff equation, from which ΔH and ΔS are estimated. **F** and **G**, Measured ΔH values and ΔS values, respectively. (n = 4-7; mean ± SEM; N.S. by one-way ANOVA).

Fig. S6. The information of plasmid carried by Lenti-virus and verification of the insertion of TRPM8 mutants.

A, Information of plasmid carried by Lenti-virus. **B,** Representative successful insertion of mTRPM8 mutant as validated by enzymatic digestion (SpeI and NotI) and DNA electrophoresis. The band in red box corresponded to mTRPM8 mutant.

Fig. S7. Viral injection sites for electrophysiological and behavioral experiments

Schematics of the corresponding brain regions in which viruses were detected. Each color represents the expression from a single mouse that was used for electrophysiological and behavioral experiments.

Fig. S1

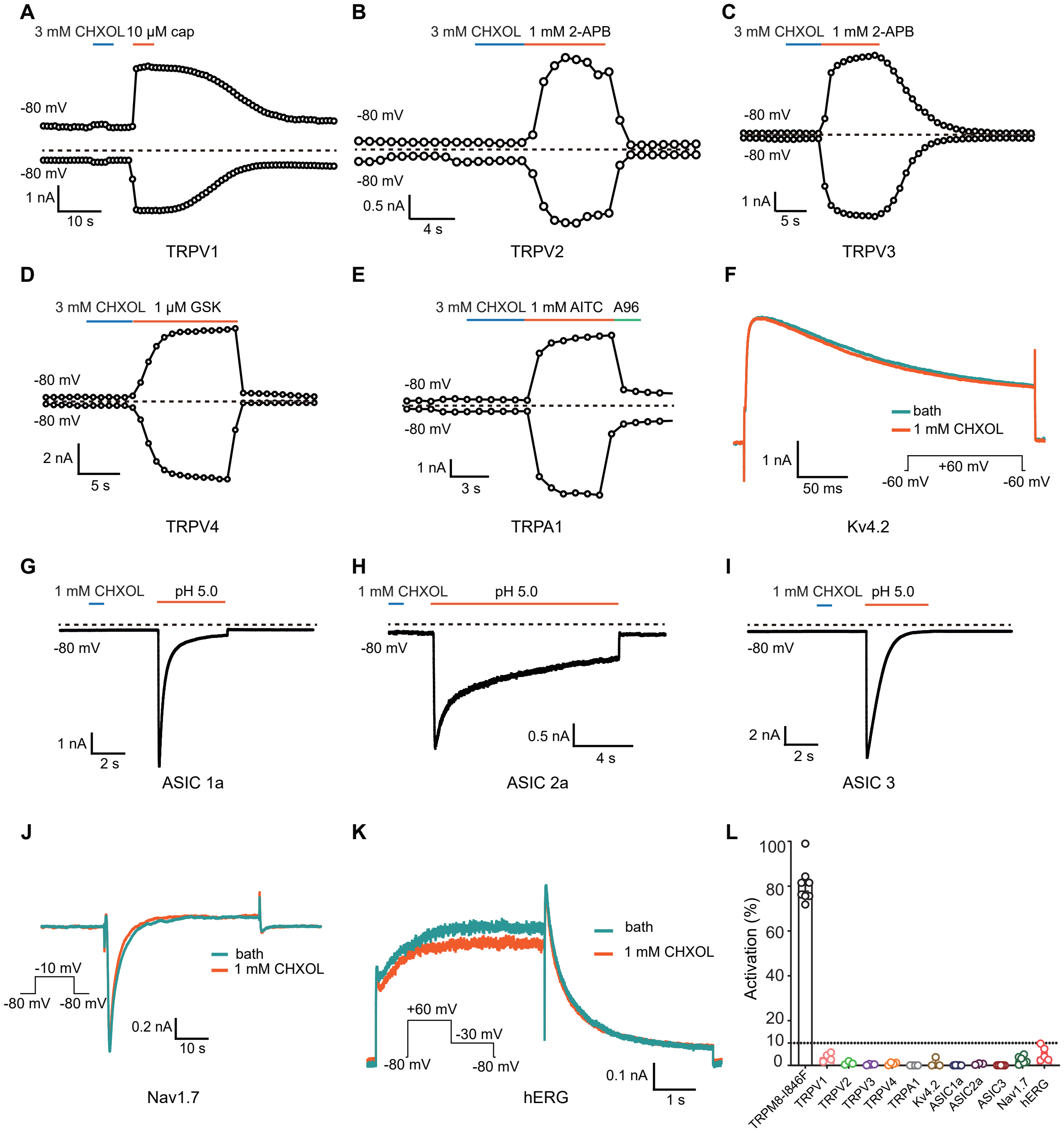
A
3 mM CHXOL 10 μM cap
-80 mV
-80 mV
1 nA
10 s
TRPV1
B
3 mM CHXOL 1 mM 2-APB
-80 mV
-80 mV
0.5 nA
4 s
TRPV2
C
3 mM CHXOL 1 mM 2-APB
-80 mV
-80 mV
1 nA
5 s
TRPV3
D
3 mM CHXOL 1 μM GSK
-80 mV
-80 mV
2 nA
5 s
TRPV4
E
3 mM CHXOL 1 mM AITC A96
-80 mV
-80 mV
1 nA
3 s
TRPA1
F
bath
1 mM CHXOL
1 nA
50 ms
+60 mV
-60 mV
-60 mV
Kv4.2
G
1 mM CHXOL pH 5.0
-80 mV
1 nA
2 s
ASIC 1a
H
1 mM CHXOL pH 5.0
-80 mV
0.5 nA
4 s
ASIC 2a
I
1 mM CHXOL pH 5.0
-80 mV
2 nA
2 s
ASIC 3
J
bath
1 mM CHXOL
-10 mV
-80 mV -80 mV
0.2 nA
10 s
Nav1.7
K
bath
1 mM CHXOL
+60 mV
-30 mV
-80 mV
-80 mV
0.1 nA
1 s
hERG
L
Activation (%)
100
80
60
40
20
10
0
TRPM8-I846F
TRPV1
TRPV2
TRPV3
TRPV4
TRPA1
Kv4.2
ASIC1a
ASIC2a
ASIC3
Nav1.7
hERG

Fig. S2

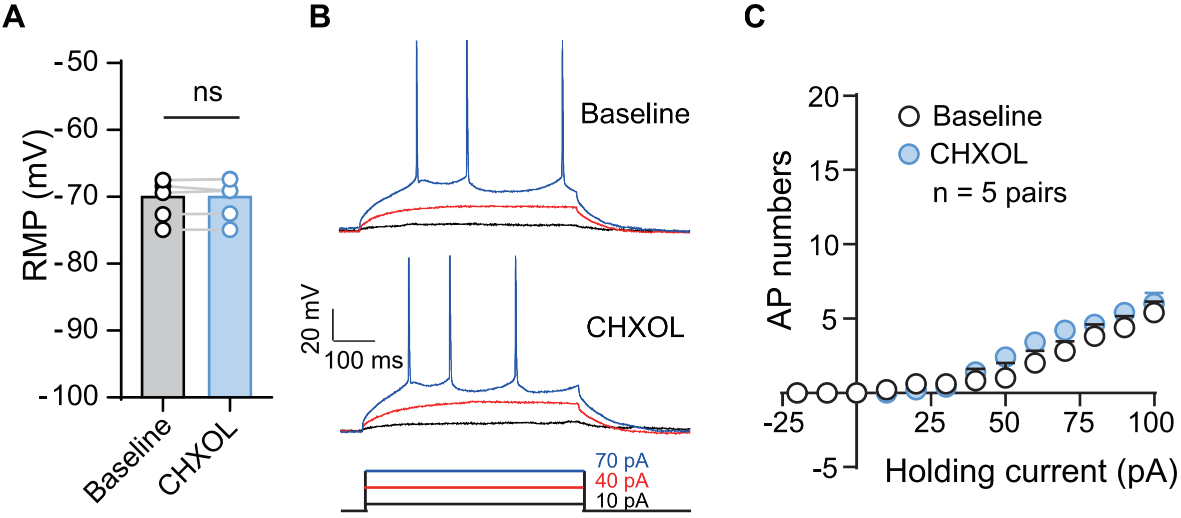

A
ns
RMP (mV)
-50
-60
-70
-80
-90
-100
Baseline
CHXOL
B
Baseline
CHXOL
20 mV
100 ms
70 pA
40 pA
10 pA
C
Baseline
CHXOL
n = 5 pairs
AP numbers
20
15
10
5
-5
-25
25
50
75
100
Holding current (pA)

Fig. S3

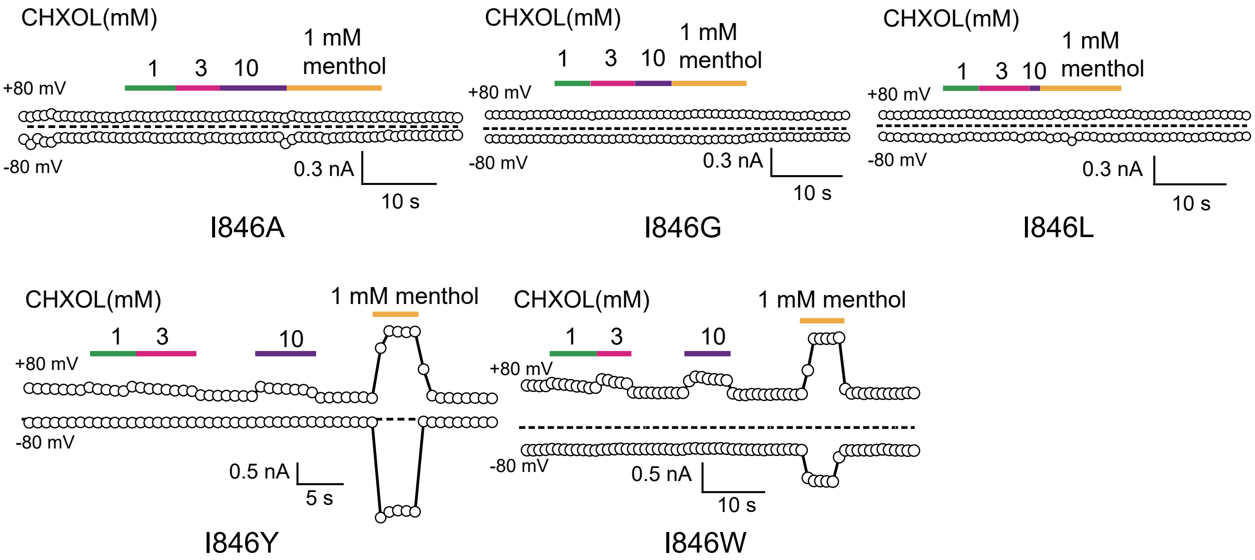
CHXOL(mM)
1 mM menthol
1
3
10
+80 mV
-80 mV
0.3 nA
10 s
I846A
I846G
I846L
0.5 nA
5 s
I846Y
I846W

Fig. S4

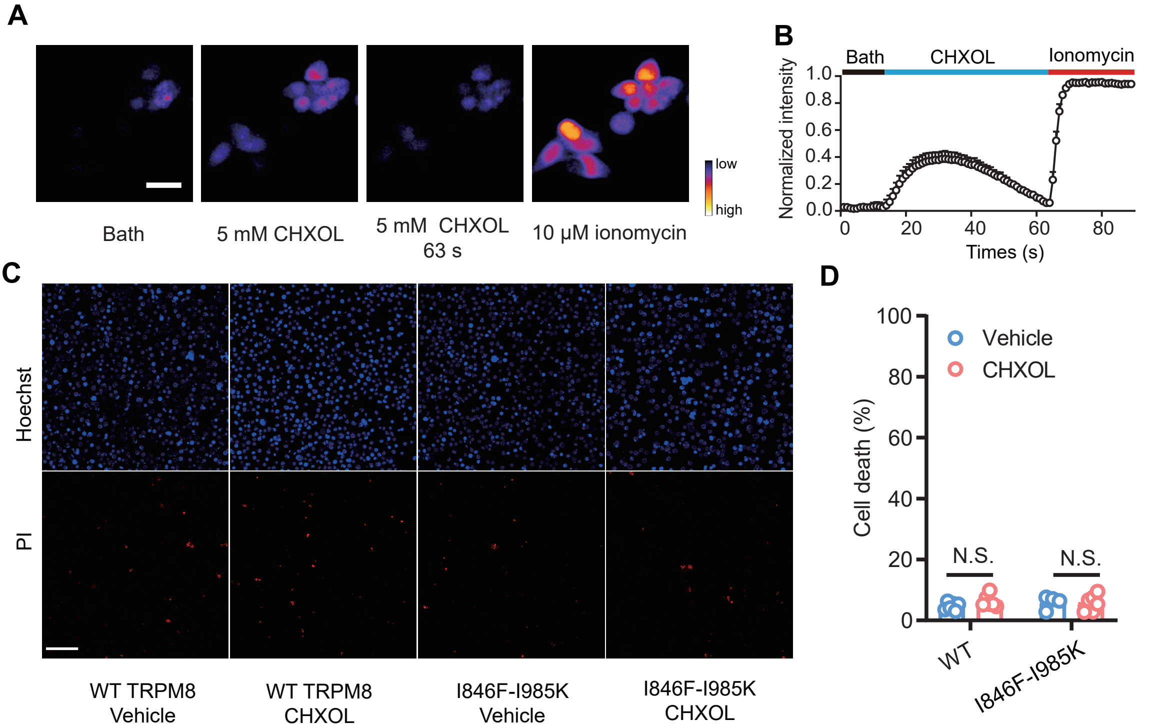

A
low
high
Bath
5 mM CHXOL
5 mM CHXOL 63 s
10 μM ionomycin
B
Bath
CHXOL
Ionomycin
Normalized intensity
1.0
0.8
0.6
0.4
0.2
0.0
0
20
40
60
80
Times (s)
C
Hoechst
PI
WT TRPM8 Vehicle
WT TRPM8 CHXOL
I846F-I985K Vehicle
I846F-I985K CHXOL
D
Vehicle
CHXOL
Cell death (%)
100
80
60
40
20
0
N.S.
N.S.
WT
I846F-I985K

Fig. S5

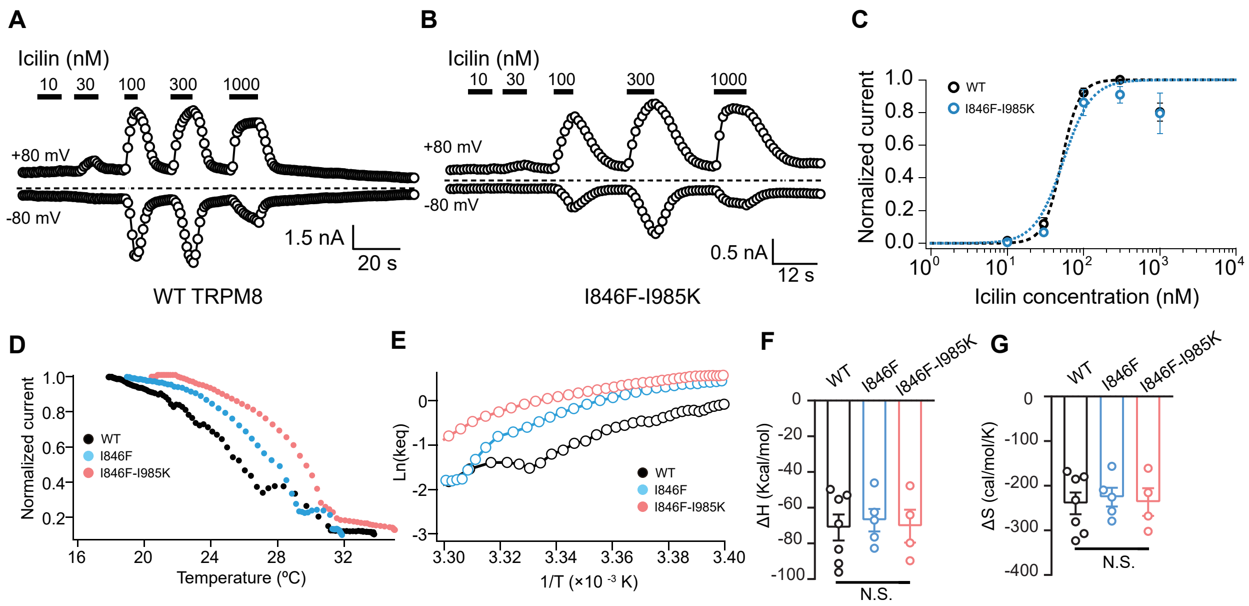
A
Icilin (nM)
10
30
100
300
1000
+80 mV
-80 mV
1.5 nA
20 s
WT TRPM8
B
Icilin (nM)
10
30
100
300
1000
+80 mV
-80 mV
0.5 nA
12 s
I846F-I985K
C
Nomalized current
WT
I846F-I985K
Icilin concentration (nM)
D
Normalized current
WT
I846F
I846F-I985K
Temperature (ºC)
E
Ln(keq)
WT
I846F
I846F-I985K
1/T (×10 -3 K)
F
WT
I846F
I846F-I985K
ΔH (Kcal/mol)
N.S.
G
WT
I846F
I846F-I985K
ΔS (cal/mol/K)
N.S.

Fig. S6

A

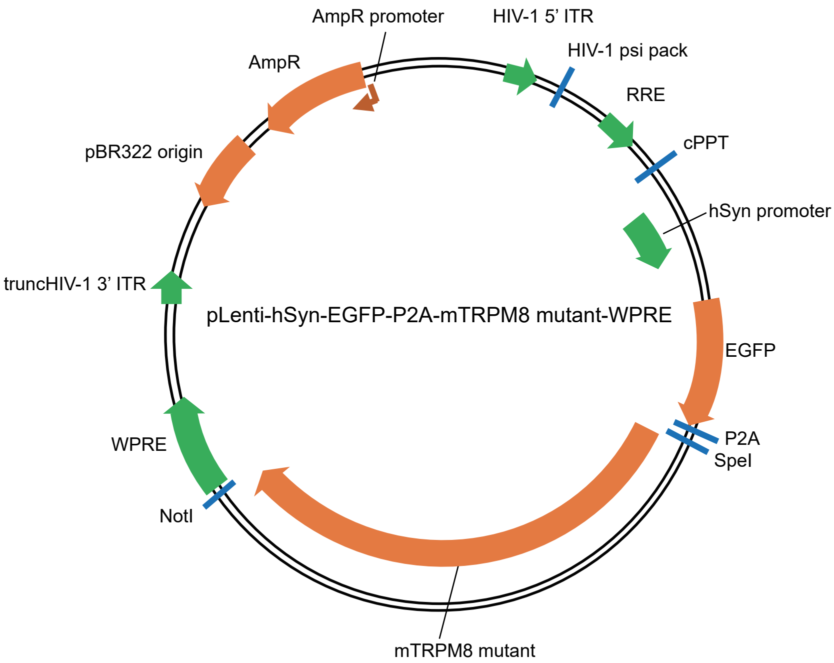
AmpR promoter
HIV-1 5' ITR
HIV-1 psi pack
AmpR
RRE
pBR322 origin
cPPT
hSyn promoter
truncHIV-1 3' ITR
pLenti-hSyn-EGFP-P2A-mTRPM8 mutant-WPRE
EGFP
WPRE
P2A
SpeI
NotI
mTRPM8 mutant

B

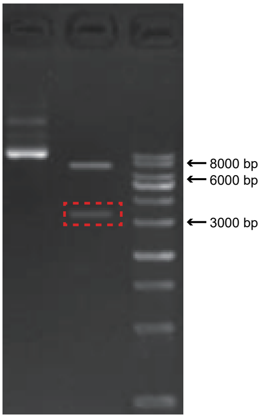
8000 bp
6000 bp
3000 bp

Fig. S7

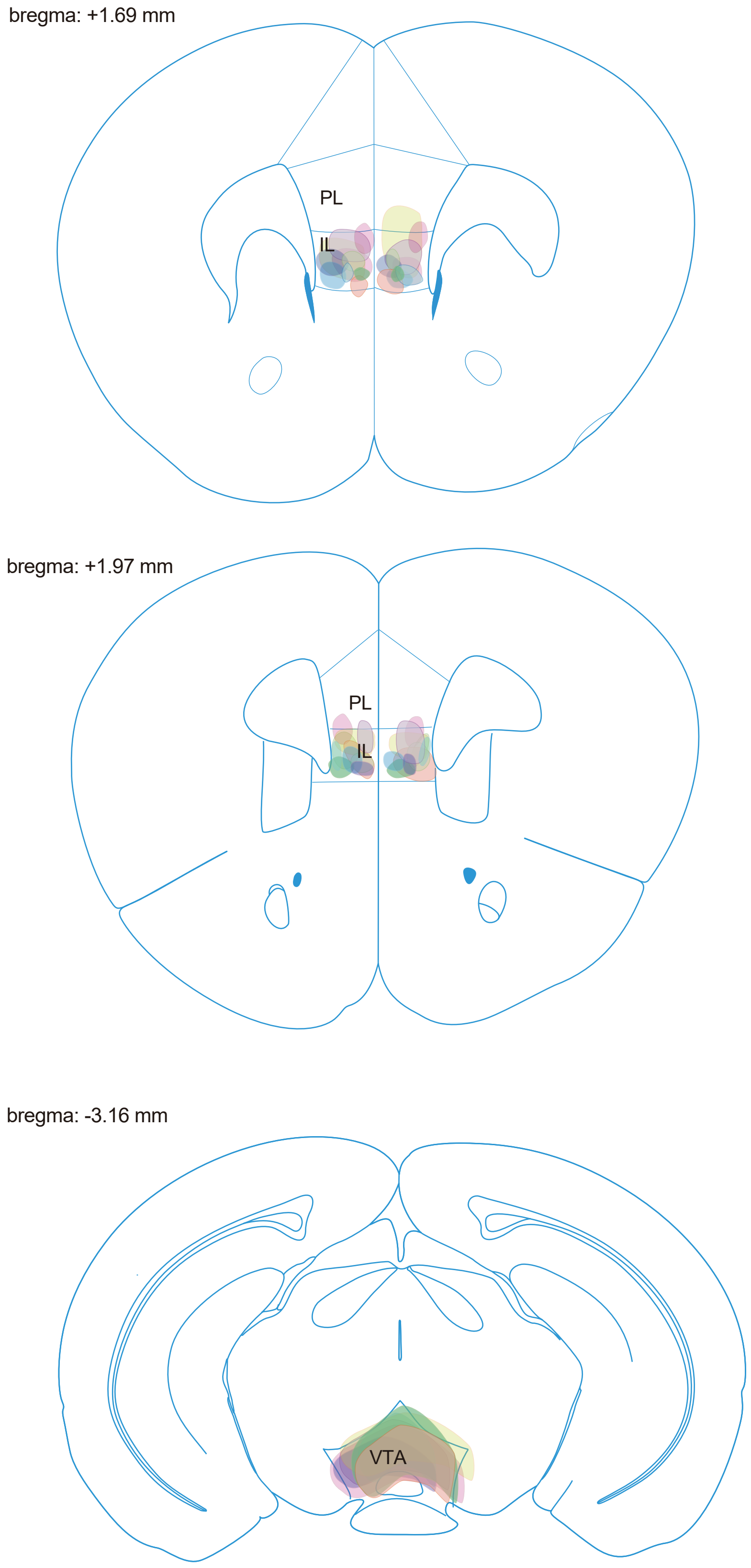

Table. S1 Expression regions of ion channels in neural system

Ion channel	Expression regions in neural system	references
TRPV1	Hippocampus; midbrain; cerebellum; cortex; olfactory bulb; spinal cord; intrafascicular nucleus; dorsomedial hypothalamus; posterior hypothalamus; DRG	(Roberts et al., 2004; Kim et al., 2005; Cavanaugh et al., 2011; Nam et al., 2015; Naziroglu and Ovey, 2015; Balleza-Tapia et al., 2018)
TRPV2	Hypothalamus; brainstem; spinal motor neurons; dorsal root ganglia; forebrain; cerebellum spinal cord trigeminal ganglia; DRG	(Shibasaki et al., 2010; Nilius and Flockerzi, 2014; Katanosaka et al., 2018)
TRPV3	Cortex; thalamus; trigeminal ganglia; superior cervical ganglia; cortex; dorsal spinal cord; dorsal root ganglia (monkey);	(Xu et al., 2002; Chen et al., 2022)
TRPV4	Hippocampus; cortex; cerebellum; hypothalamus; DRG	(Kauer and Gibson, 2009; Zhang et al., 2013; Zhang et al., 2017; Wang et al., 2019)
TRPA1	Hippocampus; DRG; trigeminal ganglion; nodose	(Nagata et al., 2005; Lee et al., 2016)
Kv4.2	hippocampus cerebellum and caudate putamen and thalamus	(Alfaro-Ruiz et al., 2019)
ASIC1a	DRG; olfactory bulb; cerebral cortex; hippocampus; habenula; basolateral amygdaloid nuclei; cerebellum	(Waldmann et al., 1997)
ASIC2a	Hippocampus Cortex; olfactory bulb; habenula; basolateral amygdaloid nuclei; cerebellum	(Lingueglia et al., 1997; Askwith et al., 2004)
ASIC3	Predominately expressed in PNS; detected in mesencephalic trigeminal neurons in the CNS	(Wemmie et al., 2013)
Nav1.7	Nociceptive DRG neurons; myenteric neurons; olfactory sensory neurons (OSNs); visceral sensory neurons; trigeminal ganglion neurons; thalamus; medial amygdala	(Toledo-Aral et al., 1997; Dib-Hajj et al., 2013; Branco et al., 2016; Kanellopoulos et al., 2018)
hERG	Particularly highly expressed in heart	(Pond et al., 2000)

Reference

Alfaro-Ruiz, R., Aguado, C., Martin-Belmonte, A., Moreno-Martinez, A.E., and Lujan, R. (2019). Expression, Cellular and Subcellular Localisation of Kv4.2 and Kv4.3 Channels in the Rodent Hippocampus. Int J Mol Sci 20.

Askwith, C.C., Wemmie, J.A., Price, M.P., Rokhlina, T., and Welsh, M.J. (2004). Acid-sensing ion channel 2 (ASIC2) modulates ASIC1 H+-activated currents in hippocampal neurons. J Biol Chem 279, 18296-18305.

Balleza-Tapia, H., Crux, S., Andrade-Talavera, Y., Dolz-Gaiton, P., Papadia, D., Chen, G., Johansson, J., and Fisahn, A. (2018). TrpV1 receptor activation rescues neuronal function and network gamma oscillations from Abeta-induced impairment in mouse hippocampus in vitro. Elife 7.

Branco, T., Tozer, A., Magnus, C.J., Sugino, K., Tanaka, S., Lee, A.K., Wood, J.N., and Sternson, S.M. (2016). Near-Perfect Synaptic Integration by Nav1.7 in Hypothalamic Neurons Regulates Body Weight. Cell 165, 1749-1761.

Cavanaugh, D.J., Chesler, A.T., Jackson, A.C., Sigal, Y.M., Yamanaka, H., Grant, R., O'Donnell, D., Nicoll, R.A., Shah, N.M., Julius, D., *et al.* (2011). Trpv1 reporter mice reveal highly restricted brain distribution and functional expression in arteriolar smooth muscle cells. J Neurosci 31, 5067-5077.

Chen, X., Zhang, J., and Wang, K. (2022). Inhibition of intracellular proton-sensitive Ca(2+)-permeable TRPV3 channels protects against ischemic brain injury. Acta Pharm Sin B 12, 2330-2347.

Dib-Hajj, S.D., Yang, Y., Black, J.A., and Waxman, S.G. (2013). The Na(V)1.7

sodium channel: from molecule to man. Nat Rev Neurosci 14, 49-62.

Kanellopoulos, A.H., Koenig, J., Huang, H., Pyrski, M., Millet, Q., Lolignier, S., Morohashi, T., Gossage, S.J., Jay, M., Linley, J.E., *et al.* (2018). Mapping protein interactions of sodium channel Na(V)1.7 using epitope-tagged gene-targeted mice. EMBO J 37, 427-445.

Katanosaka, K., Takatsu, S., Mizumura, K., Naruse, K., and Katanosaka, Y. (2018). TRPV2 is required for mechanical nociception and the stretch-evoked response of primary sensory neurons. Sci Rep 8, 16782.

Kauer, J.A., and Gibson, H.E. (2009). Hot flash: TRPV channels in the brain. Trends Neurosci 32, 215-224.

Kim, S.R., Lee, D.Y., Chung, E.S., Oh, U.T., Kim, S.U., and Jin, B.K. (2005). Transient receptor potential vanilloid subtype 1 mediates cell death of mesencephalic dopaminergic neurons in vivo and in vitro. J Neurosci 25, 662-671.

Lee, K.I., Lee, H.T., Lin, H.C., Tsay, H.J., Tsai, F.C., Shyue, S.K., and Lee, T.S. (2016). Role of transient receptor potential ankyrin 1 channels in Alzheimer's disease. J Neuroinflammation 13, 92.

Linguеglia, E., de Weille, J.R., Bassilana, F., Heurteaux, C., Sakai, H., Waldmann, R., and Lazdunski, M. (1997). A modulatory subunit of acid sensing ion channels in brain and dorsal root ganglion cells. J Biol Chem 272, 29778-29783.

Meiler, J., and Baker, D. (2006). ROSETTALIGAND: protein-small molecule docking with full side-chain flexibility. Proteins 65, 538-548.

Miteva, M.A., Guyon, F., and Tufféry, P. (2010). Frog2: Efficient 3D conformation

ensemble generator for small compounds. Nucleic Acids Res 38, W622-627.

Nagata, K., Duggan, A., Kumar, G., and Garcia-Anoveros, J. (2005). Nociceptor and hair cell transducer properties of TRPA1, a channel for pain and hearing. J Neurosci 25, 4052-4061.

Nam, J.H., Park, E.S., Won, S.Y., Lee, Y.A., Kim, K.I., Jeong, J.Y., Baek, J.Y., Cho, E.J., Jin, M., Chung, Y.C., *et al.* (2015). TRPV1 on astrocytes rescues nigral dopamine neurons in Parkinson's disease via CNTF. Brain 138, 3610-3622.

Naziroglu, M., and Ovey, I.S. (2015). Involvement of apoptosis and calcium accumulation through TRPV1 channels in neurobiology of epilepsy. Neuroscience 293, 55-66.

Nilius, B., and Flockerzi, V. (2014). Mammalian transient receptor potential (TRP) cation channels. Preface. Handb Exp Pharmacol 223, v - vi.

Pond, A.L., Scheve, B.K., Benedict, A.T., Petrecca, K., Van Wagoner, D.R., Shrier, A., and Nerbonne, J.M. (2000). Expression of distinct ERG proteins in rat, mouse, and human heart. Relation to functional I(Kr) channels. J Biol Chem 275, 5997-6006.

Roberts, J.C., Davis, J.B., and Benham, C.D. (2004). [3H]Resiniferatoxin autoradiography in the CNS of wild-type and TRPV1 null mice defines TRPV1 (VR-1) protein distribution. Brain Res 995, 176-183.

Shibasaki, K., Murayama, N., Ono, K., Ishizaki, Y., and Tominaga, M. (2010). TRPV2 enhances axon outgrowth through its activation by membrane stretch in developing sensory and motor neurons. J Neurosci 30, 4601-4612.

Smith, C.A., and Kortemme, T. (2008). Backrub-like backbone simulation

recapitulates natural protein conformational variability and improves mutant side-chain prediction. J Mol Biol 380, 742-756.

Toledo-Aral, J.J., Moss, B.L., He, Z.J., Koszowski, A.G., Whisenand, T., Levinson, S.R., Wolf, J.J., Silos-Santiago, I., Halegoua, S., and Mandel, G. (1997). Identification of PN1, a predominant voltage-dependent sodium channel expressed principally in peripheral neurons. Proc Natl Acad Sci U S A 94, 1527-1532.

Valera, S., Hussy, N., Evans, R.J., Adami, N., North, R.A., Surprenant, A., and Buell, G. (1994). A new class of ligand-gated ion channel defined by P2x receptor for extracellular ATP. Nature 371, 516-519.

Waldmann, R., Champigny, G., Bassilana, F., Heurteaux, C., and Lazdunski, M. (1997). A proton-gated cation channel involved in acid-sensing. Nature 386, 173-177.

Wang, Z., Zhou, L., An, D., Xu, W., Wu, C., Sha, S., Li, Y., Zhu, Y., Chen, A., Du, Y.*, et al.* (2019). TRPV4-induced inflammatory response is involved in neuronal death in pilocarpine model of temporal lobe epilepsy in mice. Cell Death Dis 10, 386.

Wemmie, J.A., Taugher, R.J., and Kreple, C.J. (2013). Acid-sensing ion channels in pain and disease. Nat Rev Neurosci 14, 461-471.

Xu, H., Ramsey, I.S., Kotecha, S.A., Moran, M.M., Chong, J.A., Lawson, D., Ge, P., Lilly, J., Silos-Santiago, I., Xie, Y.*, et al.* (2002). TRPV3 is a calcium-permeable temperature-sensitive cation channel. Nature 418, 181-186.

Xu, L., Zhang, H., Wang, Y., Lu, X., Zhao, Z., Ma, C., Yang, S., Yarov-Yarovoy, V., Tian, Y., Zheng, J.*, et al.* (2021). De Novo Design of Peptidic Positive Allosteric Modulators Targeting TRPV1 with Analgesic Effects. Adv Sci (Weinh) 8, e2101716.

Yang, F., Xiao, X., Cheng, W., Yang, W., Yu, P., Song, Z., Yarov-Yarovoy, V., and Zheng, J. (2015). Structural mechanism underlying capsaicin binding and activation of the TRPV1 ion channel. Nat Chem Biol 11, 518-524.

Yang, S., Lu, X., Wang, Y., Xu, L., Chen, X., Yang, F., and Lai, R. (2020). A paradigm of thermal adaptation in penguins and elephants by tuning cold activation in TRPM8. Proc Natl Acad Sci U S A 117, 8633-8638.

Zhang, H., Lin, J.J., Xie, Y.K., Song, X.Z., Sun, J.Y., Zhang, B.L., Qi, Y.K., Xu, Z.Z., and Yang, F. (2023). Structure-guided peptide engineering of a positive allosteric modulator targeting the outer pore of TRPV1 for long-lasting analgesia. Nat Commun 14, 4.

Zhang, H., Sun, X., Qi, H., Ma, Q., Zhou, Q., Wang, W., and Wang, K. (2019). Pharmacological Inhibition of the Temperature-Sensitive and Ca(2+)-Permeable Transient Receptor Potential Vanilloid TRPV3 Channel by Natural Forsythoside B Attenuates Pruritus and Cytotoxicity of Keratinocytes. J Pharmacol Exp Ther 368, 21-31.

Zhang, L., Papadopoulos, P., and Hamel, E. (2013). Endothelial TRPV4 channels mediate dilation of cerebral arteries: impairment and recovery in cerebrovascular pathologies related to Alzheimer's disease. Br J Pharmacol 170, 661-670.

Zhang, N., Yang, S., Wang, C., Zhang, J., Huo, L., Cheng, Y., Wang, C., Jia, Z., Ren, L., Kang, L., *et al.* (2017). Multiple target of hAmylin on rat primary hippocampal neurons. Neuropharmacology 113, 241-251.
